# The Effect of Stress Ratios on the Very High Cycle Fatigue Behavior of 9%Cr Turbine Steel at 630 °C

**DOI:** 10.3390/ma13163444

**Published:** 2020-08-05

**Authors:** Quanyi Wang, Yao Chen, Yongjie Liu, Chong Wang, Lang Li, Chao He, Xiufang Gong, Tianjian Wang, Wei Zhang, Qingyuan Wang, Hong Zhang

**Affiliations:** 1 School of Aeronautics and Astronautics, Sichuan University, Chengdu 610065, China; wangquanyi@stu.scu.edu.cn; 2 Failure Mechanics and Engineering Disaster Prevention and Mitigation Key Laboratory of Sichuan Province, College of Architecture and Environment, Sichuan University, Chengdu 610065, China; chenyao1101@126.com (Y.C.); liuyongjie@scu.edu.cn (Y.L.); wangchongscu@163.com (C.W.); lilang@scu.edu.cn (L.L.); hechao@scu.edu.cn (C.H.); 3 Key Laboratory of Deep Underground Science and Engineering, Ministry of Education, Sichuan University, Chengdu 610065, China; 4 State Key Laboratory of Long-Life High Temperature Materials, DongFang Turbine Co., Ltd., Deyang 618000, China; gongxiufang@dongfang.com (X.G.); wangtianjian@dongfang.com (T.W.); 5 DongFang Boiler Group Co., Ltd., ZiGong 643001, China; zhwei0916@163.com; 6 School of Architecture and Civil Engineering, Chengdu University, Chengdu 610106, China

**Keywords:** 9%Cr turbine steel, very high cycle fatigue, stress intensity factor, stress ratio, high temperature

## Abstract

Effects of the stress ratio on the very high cycle fatigue behaviors of 9%Cr turbine steel have been investigated at 630 °C. The experimental results show that the S–N curve has a continuous downward trend and has no fatigue limit with the increasing in the cycles at 630 °C. Meanwhile, according to the analysis of microstructure, there are two failure modes that were observed at different stress ratios (R = −1 and 0.1), including surface crack failure and internal crack failure, respectively. Besides, the theoretical threshold value of the crack growth is compared with the calculated value of the fracture surface. To decrease the difference between the threshold value of internal crack initiation and the corresponding theoretical value, a new model for the crack growth threshold of the interior-induced fracture at different stress ratios is proposed and discussed.

## 1. Introduction

The 9%Cr turbine steel is widely used due to the excellent ductility and superior thermal cycling properties under high temperature [1] in thermal power stations. Fatigue behaviors at high temperature are the major concerns for service. It is related to the creep-fatigue, low-cycle fatigue (LCF), and high-cycle fatigue (HCF) at the high temperature [2,3,4,5,6,7,8,9].

**Figure 1:**
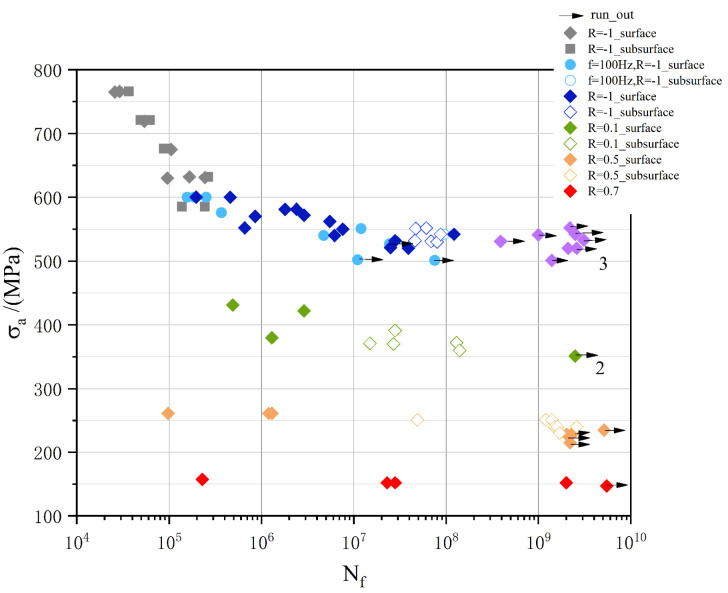
The S–N curves of Cr-based steel at room temperature (RT).

For the turbine rotor, it is found that the fatigue performance at the high temperature service section is degraded most seriously [10]. The real accident also shows that steam turbine rotors are more likely to fail at the high temperature and low-stress conditions [11], which the fatigue life falls into the very high cycle fatigue (VHCF) regime (10^9^ cycles). Therefore, it is crucial to study the VHCF behaviors of turbine steel under high temperature. Hui et al. [12] focused on the influence of hydrogen content on the behavior of Cr-based steel. Zhu et al. [13,14] mainly focused on the effect of welding efficiency, the welding process, and experimental frequency on 25Cr2Ni2MoV steel. Hong et al. [15] evaluated the fatigue life of the high carbon chromium steel containing 1.04%Cr through the fine granular area (FGA) region obtained from the VHCF test with R = −1. Zhang et al. [16] studied the failure mechanism of the welded joint of 9%Cr through the VHCF test with R = 0. Additionally, Hilgendorff [17] illustrated the cyclic deformation behavior of austenitic Cr–Ni-steels at low-stress amplitude in the VHCF strength state. However, the effect of stress ratios on the 9%Cr turbine steel is limited. Therefore, the purpose of this work is to study the influence of stress ratios on the fatigue failure behavior of 9%Cr turbine steel in the VHCF regime at 630 °C. According to the references [18,19,20], the S–N curve under different stress ratios at room temperature is shown in Figure 1. Its fatigue limit of Cr-based steel at different stress ratios is presented in Figure 1.

In this work, the high temperature ultrasonic fatigue test system is used to perform the VHCF tests at R = −1 and 0.1, respectively. Then, the scanning electron microscopy (SEM) is used to observe the microstructure of failed specimens, then to analyze the fracture modes under different stress ratios at high temperature. In the end, the fatigue failure mechanism of the whole process from the initial fracture to final fracture is discussed and deduced.

## 2. Material and Experimental Process

**Figure 2:**
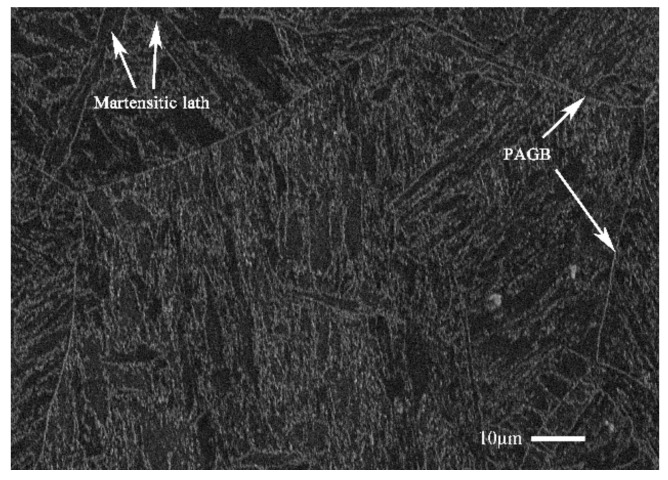
The microstructure of 9%Cr turbine steel.

The 9%Cr turbine steel was used in this study. The chemical compositions of 9%Cr turbine steel are shown in Table 1. A Vickers hardness tester (HVS-50) was used to perform the hardness test of 15 s under a maintaining load of 1 N, and the average value was 263 HV at room temperature (RT). The metallographic microstructure is presented in Figure 2, including martensitic laths and prior austenite grain boundaries (PAGB) indicated by the white arrows.

**Figure 3:**
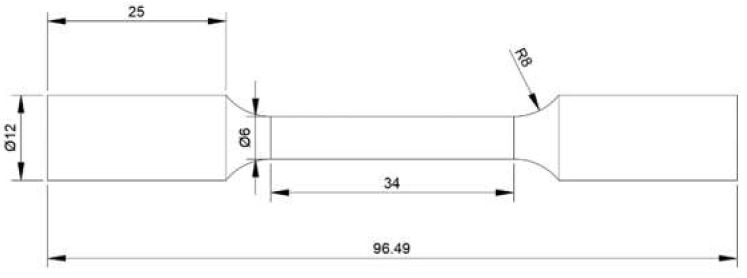
The size of the axial tensile specimen (units: mm).

The axial tensile tests with a constant strain rate of 5 × 10^−4^ s^−1^ at RT and 630 °C are performed on a 100 kN SHIMADZU tensile test machine. A high temperature furnace with thermocouples was used to heat the tensile sample, while a high temperature extensometer with a gauge length of 25 mm was used to control and measure strain, where the temperature fluctuation was less than ±2 °C. The axial tensile specimens are presented in Figure 3, and its gauge length was 34 mm.

**Figure 4:**
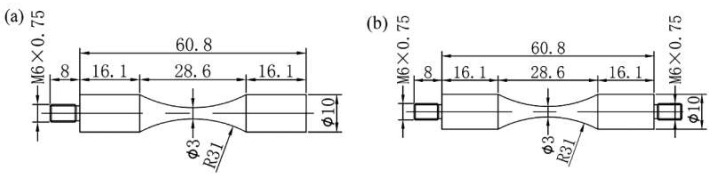
The size of the VHCF specimen (units: mm) [21], (**a**) R = −1 and (**b**) R > 0.

The VHCF specimens with the hourglass shape [21] are shown in Figure 4, in which the diameter of both specimens was 3 mm. In this study, the fatigue tests were cycled until 10^9^ cycles with the constant frequency of 20 kHz at R = −1 and 0.1, respectively. The self-developed electromagnetic heating equipment was used to reach the target temperatures with a fluctuation range of ±5 °C. To reduce the self-heating of the VHCF specimens, the intermittent vibration loading was used, where the vibration time and rest time were 100 ms and 300 ms, respectively.

**Figure 5:**
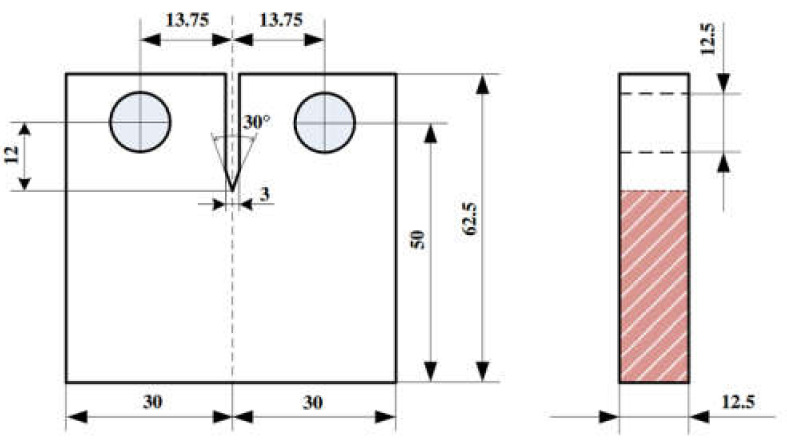
The size of fatigue crack growth (CT) specimen (units: mm) [23].

To investigate the fatigue crack growth (FCG) of 9%Cr turbine steel at 630 °C, the compact tension (CT) specimens were designed following ASTM E647 (see Figure 5) [22]. The high temperature furnace was used to control temperature fluctuation within ± 2 °C at R = 0.1 and loading frequency was 10 Hz. Before FCG tests, specimens were preheated up to 630 °C for 10 min.

## 3. Results and Discussion

### 3.1. The Tensile Behavior

**Figure 6:**
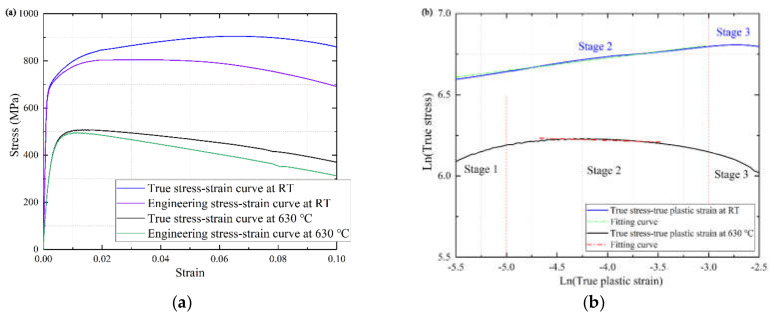
The true stress–strain curves of 9%Cr turbine steel at RT and 630 °C. (**a**) monotonic stress-strain curves; (**b**) strain hardening exponent fitted by Equation (1).

The basic tensile performances of 9%Cr at different temperatures are shown in Figure 6a. Comparison of the RT, the tensile softening behavior of 9%Cr at 630 °C are presented. Additionally, there was no obvious strengthening stage at 630 °C where the stress quickly reached the ultimate tensile strength exceeded the yield stress. Moreover, the empirical formula was served to appraise the behavior of the strain hardening and get the crucial parameters by Equation (1).

(1) $$\sigma {=K}_{1}\varepsilon_{p}^{n_{1}}$$

where σ is the true stress, $\varepsilon_{p}^{n_{1}}$ is the true plastic strain, and K_1_ and n_1_ are the material strength parameters and strain hardening exponent, respectively. Equation (1) was used to fit the double logarithmic plot of the true stress–true plastic strain, as shown in the Figure 6b. The true plastic behavior has a clear difference between RT and 630 °C. The true plastic curve of RT was higher than that of the high temperature, this presents the softening behavior at 630 °C was more serious than that at RT. Additionally, the hardening curve of RT can be split into two stages, i.e., ascending stage 2 and descent and failure stage 3. The hardening curve of 630 °C can be divided into three regions, i.e., ascending stage 1, steadily drop stage 2, and failure stages 3. It indicates that high temperature has a great influence the softening behavior and plastic flow of 9%Cr during the uniaxial tensile test. Then, the slope of phases is fitted by the Equation (1) to get the strain hardening exponent. More detail parameters are presented in Table 2.

### 3.2. S–N Curves

**Figure 7:**
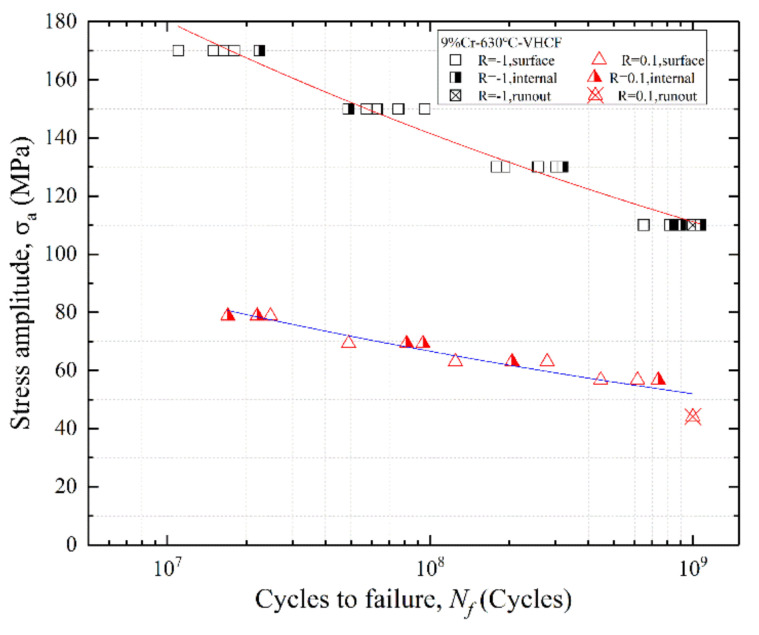
The S–N curves of 9%Cr turbine steel at R = −1 and 0.1 under 630 °C.

The S–N curves at R = −1 and 0.1 are shown in Figure 7, where the surface failure is indicated by the open marks, and the internal failure is marked by the semi-solid marks. The two S–N curves both show a continuous downward trend and the curves have not the characteristic of duplex or stepwise. Additionally, the fatigue test results of each stress amplitude were relatively concentrated. There was no traditional fatigue limit at both stress ratios. The trend of stress amplitude decreasing at R = 0.1 was significantly slower than R = −1.

There is not an apparent transition region of crack initiation from the surface-induced fracture to the interior-induced fracture from 10^7^ to 10^9^ cycles. Three kinds of fatigue crack initiation modes are observed, i.e., surface initiation, internal inclusions, and internal micropores. Most of the fatigue failure specimens at R = −1 were the surface failure, as shown in Figure 7. However, there was an internal crack initiation in every stress amplitude. Meanwhile, it is worth noting that the trend of S–N curves formed by the fatigue results of internal crack initiation and the fatigue results of surface crack initiation was similar. It indicates the modes of crack initiation had no decisive influence on the fatigue life of the specimens.

There is an agreement with the other studies [24,25], resulting from the mechanical degradation at high temperature, and the oxidation can reduce the strength of the specimen surface. Thus, the VHCF specimens are more likely to cause failure on the specimen surface at 630 °C.

### 3.3. Microstructure Characterization

**Figure 8:**
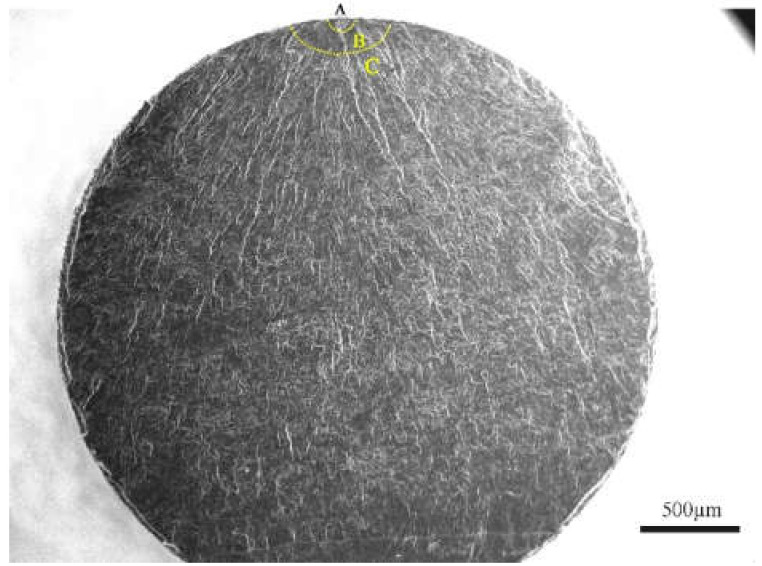
Overview of the fracture surface, R = −1, σ_a_ = 150 MPa, *N*_f_ = 6.24 × 10^7^ cycles.

Generally, the fatigue crack growth process includes crack initiation, crack propagation, and the final fracture. In order to understand the fatigue failure process of 9%Cr turbine steel at R = −1 and 0.1, three regions of fracture morphology can be marked by the two dashed lines, as shown in Figure 8 [26]. Region (A) is the crack initiation stage. Region (B) and (C) is the slow crack growth stage and the rapid crack growth, respectively.

**Figure 9:**
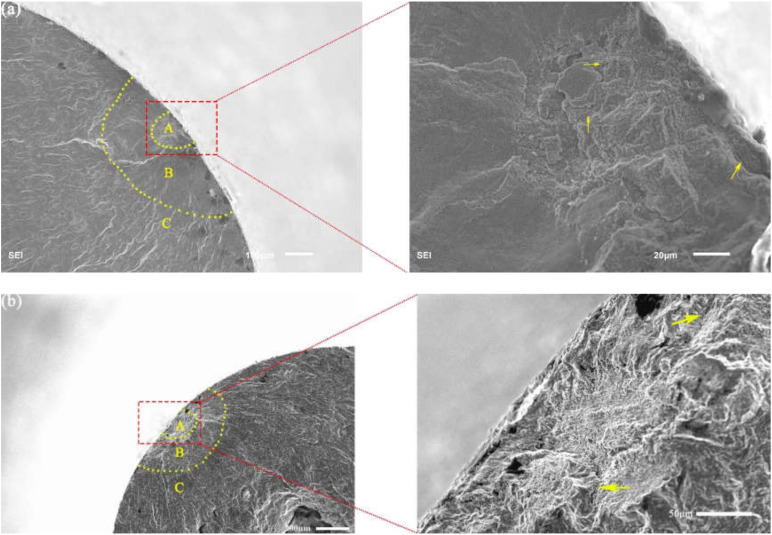
Typical fracture morphology of failure specimen with surface-induced. (**a**) R = −1, σ_a_ = 130 MPa, *N_f_* = 2.59 × 10^8^ cycles and (**b**) R = 0.1, σ_a_ = 63 MPa, *N_f_* = 2.79 × 10^8^ cycles.

The microstructure of the failure specimens with surface crack initiation under different stress ratios at 630 °C is presented in Figure 9. A high magnification image of region (A) is displayed at right side of each image. Whereas, some secondary cracks can be observed in Figure 8, as shown by the yellow arrows. Three regions were divided by two dashed lines on the whole fracture surface. The first region (A) was the region of fatigue crack initiation, which was a rough porous region without any facets like the previous studies [27,28]. There were some thick radial ridges in the second region (B), i.e., the crack propagation regime from the first region (A) and the micromorphology was relatively flat [26]. For region (C), i.e., crack rapid propagation region, there were too many slight radial ridges along the direction of primary crack propagation.

**Figure 10:**
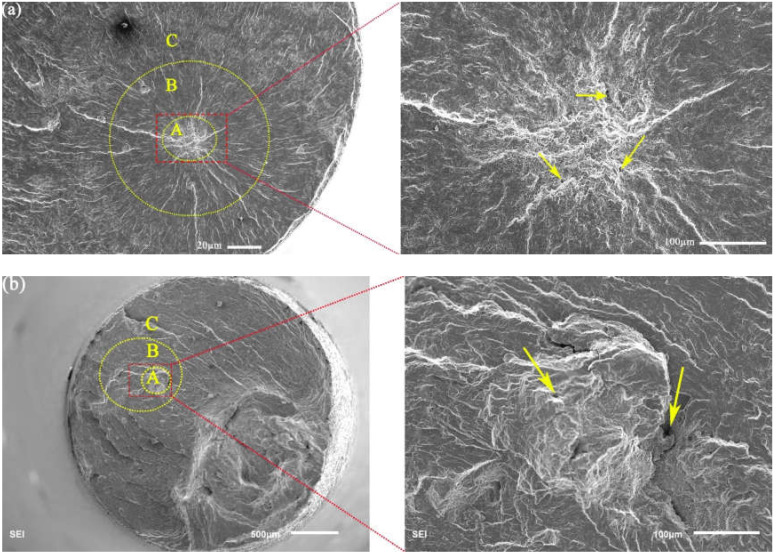
Typical fracture morphology of the failure specimen with internal cracks. (**a**) R = −1, σ_a_ = 130 MPa, *N_f_* = 1.93 × 10^8^ cycles and (**b**) R = 0.1, σ_a_ = 63 MPa, *N_f_* = 8.14 × 10^7^ cycles.

The fracture morphologies of internal failures at R = −1 and 0.1 are presented in Figure 10. The high-magnification images of region (A) are shown in the right side of each image. For R = −1, a clear fish-eye structure can be observed [29] from Figure 10a. The fish-eye structure was inside the fracture surface of the failure specimens and did not contact the edge of the failure specimens. The fracture morphology of R = −1 can also be divided into three regions, like a surface-induced fracture. Inside the fish-eye structure, there was an inclusion including the loose and porous indicated by the yellow arrow in Figure 10a. There are many thick radial ridges around inclusions, which are found on the fracture surface. The prominent fine granular area (FGA) [30] region was observed around the inclusions, and fatigue fracture was caused by the transgranular crack propagation.

However, Figure 10b shows that the fracture microstructure of internal crack initiation at R = 0.1, there were litter inclusions near the crack initiation source, and the crack initiation was caused by the micropores. Meanwhile, the characteristics of fish-eye were not observed on the test specimens of R = 0.1, and the obvious fracture microstructure caused by the tensile stress can be observed. In this study, the above three regions were divided according to the direction of crack growth by two dashed lines for R = 0.1.

In this study, there were many relatively large micropores in the positions indicated by the yellow arrows in Figure 10b, which were not found in other SEM images of R = −1. As mentioned above, the mechanical properties of this material were seriously degraded at 630 °C, as shown in Figure 6. Thus, considering the influence of the mechanical properties at 630 °C and the mean stress, the fracture section of the failure specimens must be affected by the micropores caused by the mean stress. Additionally, in the process of the experiment, the local deformation of the defect is incongruous, which leads to the local stress concentration [27]. Thus, the internal micropores easily cause crack initiation under mean stress and high temperature. It also indicates that the mean stress can speed up the formation of internal micropores marked by the yellow arrows due to the degradation of 9%Cr turbine steel under the high temperature environment. Thus, the mean stress amplitude and high temperature are the critical factors to lead to failure in the VHCF regime.

In this study, Figure 9 presents the typical fracture morphology of the surface-induced specimens at 630 °C. The typical fatigue characteristic of R = −1 can be observed at 630 °C, it is the crack initiation caused by inclusions and cracks that propagate through the crystal. However, Figure 10b shows that region (B) of the crack growth did not have the typical fatigue characteristics in region (B) for R = 0.1. Meanwhile, there were relatively large micropores in the positions indicated by the red arrows in Figure 10b, and the micropores were not found in the SEM images of R = −1 under high temperature. Thus, under the high temperature environment, the mean stress could cause the formation of the many micropores in the material and accelerate the fatigue failure process of the material.

To sum up, because the high temperature had a great influence on the mechanical properties and the oxide layer, the specimen often failed on the surface for R = −1. For R = 0.1, because the influence of mean stress and the degradation of mechanical properties at high temperature, the inside of the samples had some micropores. This possibility of internal failure of the test piece was increased because of the influence of micropores. Meanwhile, the local deformation of the micropore was incongruous, which leads to the local stress concentration [27]. So, the probability of internal failure of the specimen of R = 0.1 was higher than that of R = −1.

### 3.4. Fractography and Fracture Mechanics

**Figure 11:**
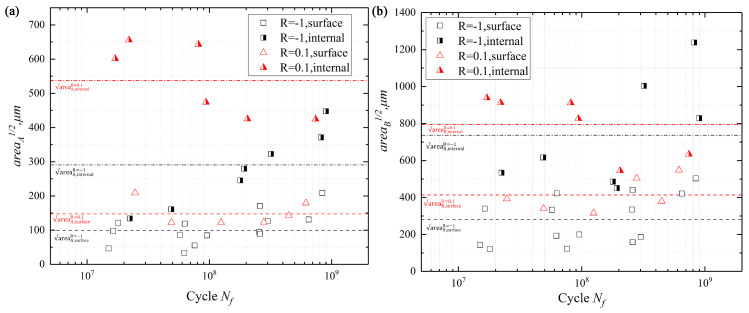
The area of the region (**A**) and (**B**) vs. fatigue life at different stress ratios.

As shown in Figure 11a, the $\sqrt{area}$ about region (A) and (B) of Figure 8 is presented in Figure 11. The relationship between $\sqrt{area}$ of region (A) and fatigue life at R = −1 and 0.1 is presented in Figure 11a, respectively. For R = −1, the area of region (A) increased with the fatigue life increasing. However, for R = 0.1, it was different, i.e., crack initiation occurred on the surface. The tendency increased with fatigue life increasing. For the interior-induced model in Figure 11a, the area of region (A) decreased with an increase in fatigue life. As shown in Figure 11b, the relationship between $\sqrt{area}$ of region (B) and fatigue life at different stress ratios is given. There was no obvious difference in the trend between the area of region (A) and fatigue life, and between the area of region (B) and fatigue life. Meanwhile, the area of internal cracks was larger than the area of surface cracks, as shown in Figure 11. It indicates that the different stress ratios could cause different failure mechanisms for two regions. For R = −1, the stress amplitudes and the inclusion size were the critical factors to control the crack initiation. For R = 0.1, the mean stress was the crucial factor of crack initiation. In the following section, the stress intensity factor (SIF) ranges (ΔK) with region (A) and (B) will be discussed.

**Figure 12:**
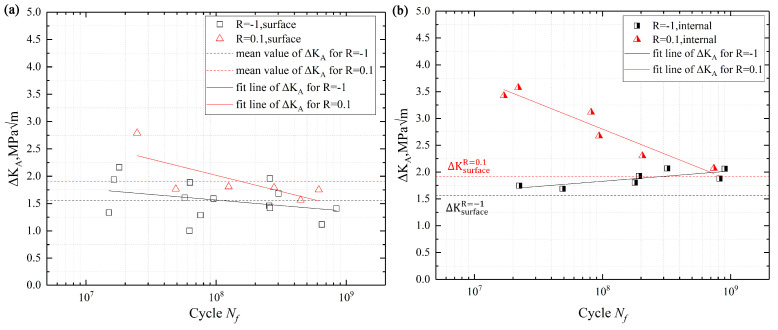
The Δ*K_A_* vs. the fatigue life: (**a**) surface initiation and (**b**) internal initiation.

In this study, the geometric parameter $\sqrt{area}$ of the region (A) was used to calculate the ΔK of the crack tip. Equation (2) of Δ*K_A_* [31,32] is shown as follows,

(2) $$\Delta K_{A}=n\Delta \sigma \sqrt{\pi \sqrt{area_{A}}}$$

where n is a constant related to the location of crack initiation, and n is 0.65 for surface-induced and 0.5 for interior-induced crack, respectively. Δσ is the applied cyclic stress range, it can be obtained from the S–N curves. $\sqrt{area}$ stands for the crack projection area, and it is marked with dotted lines in Figure 9 and Figure 10, respectively, then computed in Figure 11. In this study, because of the influence of the crack closure effect [33], the compressive stress was useless for the fatigue crack growth, Δσ only considered the range of tensile stress. Thus, the value of ΔK_A_ was calculated by Equation (2) and presented in Figure 12.

For R = −1, according to the Tayler expression [34], the yield strength and grain size were used to get a simple expression for calculating the crack threshold as follows,

(3) $$\Delta K_{\mathrm{th}}=\sigma_{y}\sqrt{\frac{2.82\pi d}{1-\nu^{2}}}$$

where, $\sigma_{y}$ is the yield strength with unit: MPa, v is the Poisson’s ratio, and d is the grain size. $\Delta K_{th}$ is the effective crack propagation threshold, unit: $MPa\sqrt{m}$. The width of martensitic lath can be obtained from the previous study [27], v = 0.3, d = 1 µm, $\sigma_{y}=443\;MPa$. Thus, $\Delta K_{th}$ = 1.38 $MPa\sqrt{m}$ in theory at R = −1.

For R = 0.1, a correlation [35] between elastic modulus (E) and effective crack growth threshold ($\Delta K_{th}$) is shown as follows,

(4) $$\Delta K_{\mathrm{th}}=\frac{3}{4}E\sqrt{\left|b\right|}$$

where, *E* is the elastic modulus, unit: MPa; |b| is the Burger’s vector, its range is 0.25–0.29 nm; and $\Delta K_{th}$ is the effective crack propagation threshold, unit: $MPa\sqrt{m}$. Thus, the calculated effective crack propagation threshold $\Delta K_{th}$ was 1.84 $MPa\sqrt{m}$ in theory at R = 0.1.

The stress intensity factor, Δ*K_A_,* which is calculated by Equation (2), is usually used to evaluate crack initiation and propagation behavior [33,36]. Meanwhile, some studies indicated the value of Δ*K_A_* at the front of FGA is almost a constant, which can correspond to the threshold of crack growth $\Delta K_{th}$ [37]. It indicated that the value of Δ*K_A_* is reasonable to evaluate the crack initiation stage. Figure 11 shows that the calculated range of ΔK*_A_*, and it indicates that the mean value of Δ*K_A_* had no apparent difference between R = −1 and R = 0.1 when the crack source was on the surface. For the internal crack initiation, its mean value of Δ*K_A_* at R = 0.1 was higher than R = −1, and the value of Δ*K_A_* at R = 0.1 decreased with the fatigue life increasing until its value was close to the result of R = −1.

For R = −1, there was no obvious relationship between the change of Δ*K_A_* on surface-induced and fatigue life, and Δ*K_A_* was a constant (1.56 $MPa\sqrt{m}$). For the interior-induced of R = −1, the mean value of Δ*K_A_* was 1.88 $MPa\sqrt{m}$. For R = 0.1, the value of Δ*K_A_* on surface-induced was a constant (1.8 $MPa\sqrt{m}$), and the mean value of Δ*K_A_* on interior-induced was 2.86 $MPa\sqrt{m}$.

Table 3 presents the theoretical crack growth threshold value $\left(\Delta K_{th}\right)$ and the mean value of Δ*K_A_*. For the surface-induced fracture, there was no significant difference between the $\Delta K_{th}$ of theoretical calculation and the experimental data. However, for the interior-induced fracture, there was a clear difference between the value of theoretical calculation and the experimental data for R = −1 and 0.1.

For the surface-induced fracture and interior-induced fracture, the main difference was the location of crack initiation. Additionally, the different crack initiation positions corresponded to different physical environments, the cracks were initiated in the air for the surface-induced fracture and the cracks were initiated in the vacuum for the interior-induced fracture. Meanwhile, the previous studies [21,38,39] indicated the local mechanical property of the crack tip is different in air and vacuum. Thus, for the interior-induced fracture of R = −1 and 0.1, the clear difference of the interior-induced fracture between the theoretical crack growth threshold value $\left(\Delta K_{th}\right)$ and experimental value is observed in Table 3, because the local mechanical properties of the crack tip are different in physical environments. Thus, a new theoretical equation was proposed based on the previous studies for the interior-induced fracture in the next section for R = −1 and 0.1.

The relationship between Δ*K_A_* and Δ*K_th_* has been mentioned above. For region (B) in Figure 9, it was the crack growth stage. In order to understand how the stress ratios affect the physical nature of the crack growth region (B), the fracture mechanics were used to evaluate the fatigue property at different stress ratios. The SIF of the region (B) [40] was calculated as follows,

For the surface-induced crack:

(5) $$\Delta K_{B}=F\Delta \sigma \sqrt{\pi b}$$

where

(6) $$F=0.61+0.413\left(\frac{b}{R}\right)-0.649{\left(\frac{b}{R}\right)}^{2}+0.838{\left(\frac{b}{R}\right)}^{3}$$

Here, Δσ stands for the tensile stress ranges, and b is the depth of crack tip from the surface. *R* is the sample radius and *R* was 1.5 mm in this study.

For the internal-induced cracks:

(7) $$\Delta K_{B}=[F_{P}\Delta P+F_{M}\frac{4\Delta Ma}{\left(R^{2}+a^{2}\right)}]\frac{\sqrt{\frac{c}{R}}}{\pi (R^{2}-a^{2})}\sqrt{\pi a}$$

where

(8) $$F_{p}=\frac{2}{\pi }\left[1+\frac{1}{2}\lambda -\frac{5}{8}\lambda^{2}\right]+0.268\lambda^{3},\lambda =\frac{a}{R}$$

where Δ*P* is the range of applied force, it can be calculated from the S–N curves, *R* is the sample radius, a is the crack radius, which can be obtained from Figure 11b, and *c* = *R* – *a*, and *M* is bending moment and the *M* was 0 due to no bending moment. The Δ*K_B_* just considers the tensile stress.

**Figure 13:**
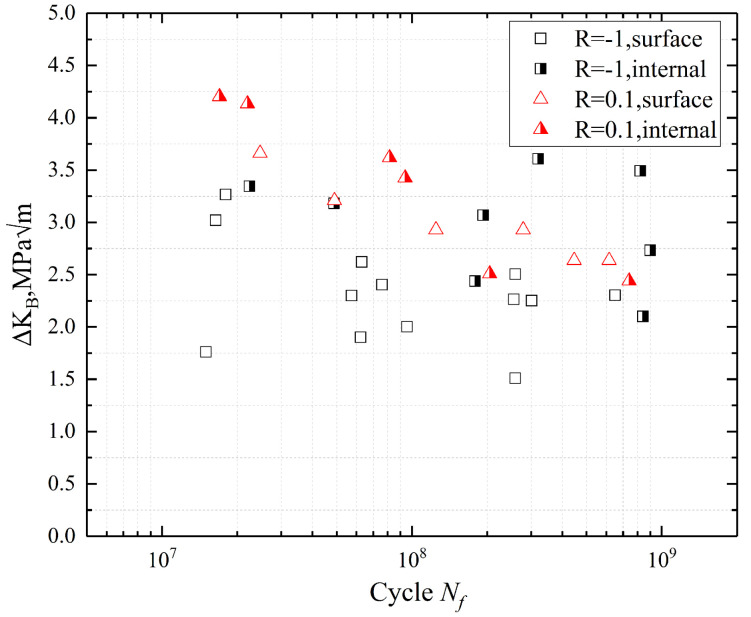
The Δ*K_B_* vs. the fatigue life.

Figure 13 shows the range of SIF for regions (B) with R = −1 and 0.1. For R = −1, the range of Δ*K_B_* was a constant (2.32 $MPa\sqrt{m}$), and it had no obvious relationship with fatigue life. However, for R = 0.1, the value of Δ*K_B_* of the surface crack and internal crack decreased with fatigue life increasing. This indicates the tensile stress could influence of the SIF of region (B) on the crack growth, and the smaller the tensile stress, the closer the SIF of region (B) was to the SIF without tensile stress.

**Figure 14:**
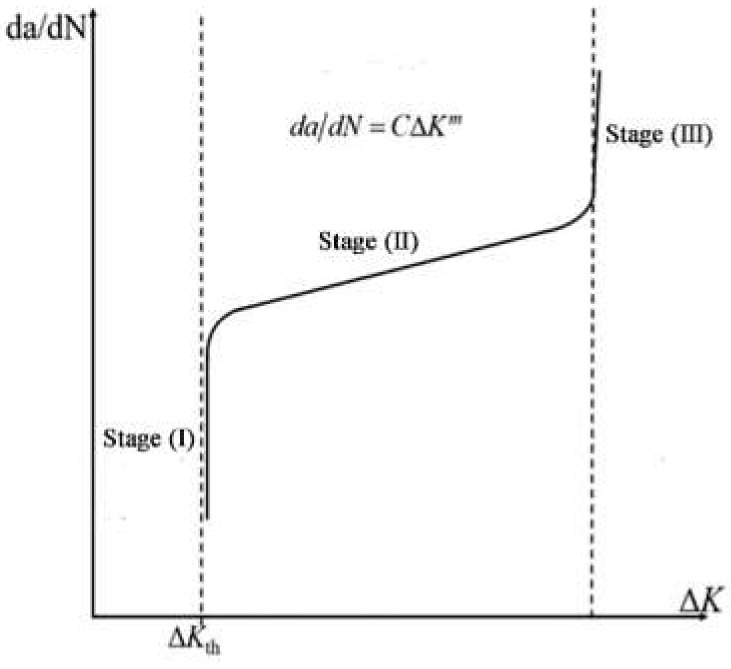
The diagram of the crack growth rate [42].

The previous researchers have shown that the SIF in the crack initiation region (A) (see Figure 8) is relatively close to the threshold value of crack growth [21,26,41], as the stage (I) in Figure 14. This indicates that the crack will enter the Pairs region when Δ*K* exceeds the crack growth threshold, which conforms to the Pairs formula, as stage (B) in Figure 14, where the Pairs formula can represent the value of Δ*K_B_*.

For the curve of the crack growth rate in the Pairs region, “$K_{max}-K_{min\;}$” and “$K_{max}-K_{op}$” can be used to describe the curves of the crack growth rate, respectively. For the method of “$K_{max}-K_{min\;}$”, it is a traditional description method for the curve of the fatigue crack growth rate. However, the method of “$K_{max}-K_{min\;}$” does not consider the influence of crack closure effect [43]. For the method of “$K_{max}-K_{op}$”, it considered the influence of the crack closure effect and presented the crack closure stress intensity factor (U) as a description of the degree of crack closure [44].

(1): “$K_{max}-K_{min\;}$” uses the stress intensity range (Δ*K*) of experimental data as the abscissa [43].

(2): “$K_{max}-K_{op}$” uses the effective stress intensity range $\left({\Delta K}_{eff}\right)$ as the abscissa [44].

The relationship between ${\Delta K}_{eff}$ and Δ*K* is as follows [44]:

(9) $$\Delta K_{\mathrm{eff}}=(0.5+0.4R)\Delta K,0.1\leq R\leq 0.5$$

**Figure 15:**
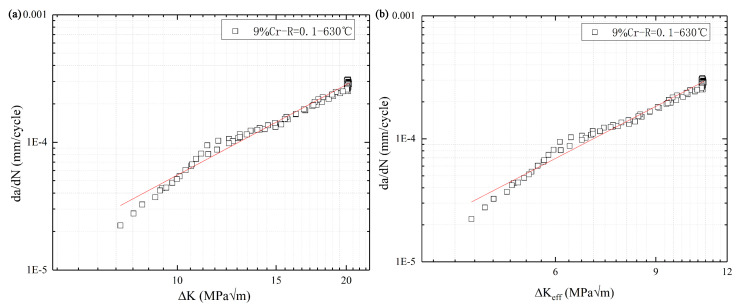
The curves of crack growth rate with R = 0.1 of 9%Cr; (**a**): $K_{max}-K_{min\;}$ and (**b**): $K_{max}-K_{op\;}$.

Figure 15 shows the curves of the crack growth rate of 9%Cr turbine steel at R = 0.1. Paris criterion presented in Equation (10) was used to describe the trend of crack growth rate in the range of low and medium stress intensity.

(10) $$\frac{\mathrm{da}}{dN}=C{\left(\Delta K\right)}^{m}$$

here, *c* and *m* are a constant, the value of c and m can be obtained from the curve fitting.

The fitting formulas of the Pairs region obtained by the method “$K_{max}-K_{min\;}$” and the “$K_{max}-K_{op}$” are presented in Equations (11) and (12), respectively.

(11) $$\frac{\mathrm{da}}{dN}=(2.15{)}^{-7}{\left(\Delta K\right)}^{2.36}$$

(12) $$\frac{\mathrm{da}}{dN}=(9.3{)}^{-7}{\left(\Delta K\right)}^{2.405}$$

**Figure 16:**
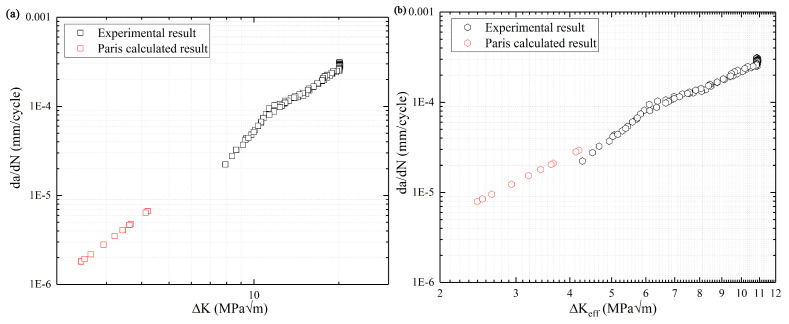
The calculated value of da/dN corresponding to the Δ*K_B_* of R = 0.1. (**a**): $K_{max}-K_{min\;}$ and (**b**): $K_{max}-K_{OP\;}$.

The calculated value of *da/dN* corresponding to the Δ*K_B_* of R = 0.1 is presented in Figure 16. It has an obvious representation in Figure 16, the method of “$K_{max}-K_{OP\;}$” has a better match with the SIF of region (B) because the effect of crack closure is eliminated, consistent with previous studies [21,26]. Additionally, the range of *da/dN* about the Δ*K_eff_* is from 10^−4^ to 10^−5^ mm/cycle, and it is in the range of Pairs region.

### 3.5. The Model of the Crack Growth Threshold (ΔK_th_) for the Interior-Induced Fracture

The previous studies [34] indicated the $\Delta K_{th}$ was related to the yield stress (*σ_y_*), grain size (*d*), and the Poisson’s ratio (*v*), as shown the Equation (3). However, as mentioned above, it has a clear difference between the theoretical threshold of crack propagation and the experimental value for the interior-induced fracture. Thus, in order to overcome the limitation of the empirical formula of the crack threshold (Δ*K_th_*) about the interior-induced fracture, a crack threshold formula about the interior-induced fracture was proposed based on Equation (3) for R = −1 and 0.1.

(13) $$\Delta K_{th,\mathrm{internal}}=\alpha \sigma_{y,\mathrm{internal}}\sqrt{\frac{2.82\pi d}{1-\upsilon^{2}}}$$

here, $\sigma_{y,\;\mathrm{internal}}$ is the local yield stress of the interior-induced fracture at the crack tip, α is the parameter about stress ratios.

Furthermore, the grain size was similar to that of the cycle plastic zone ($\gamma_{p}$) at the crack tip where is the transition of fracture morphology [45] and crack growth rate data [46]. Additionally, the local yield stress of the crack tip could be calculated by the cycle plastic zone ($\gamma_{p}$) in air and vacuum. The relationship between $\gamma_{p}$ about the model-Ι crack under the plane strain condition is as follows:

(14) $$\gamma_{p}=\frac{1}{6\pi }{\left(\frac{\Delta K_{B}}{\sigma^{y}}\right)}^{2}$$

where $\sigma_{y}$ is the yield stress.

**Figure 17:**
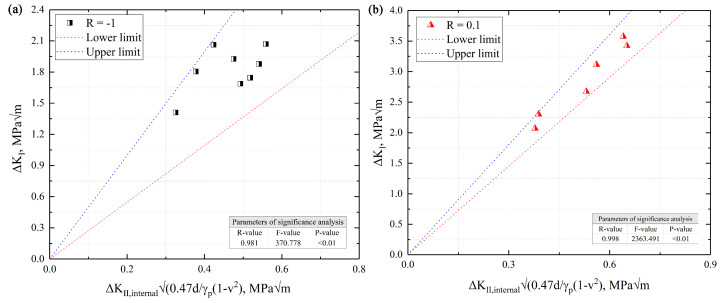
The 90% prediction interval about Equation (15) of internal-induced fracture at R = −1 and 0.1. (**a**) the statistical analysis of the interior-induced for R = –1; (**b**) the statistical analysis of the interior-induced for R = 0.1.

The above discussion indicates the values of Δ*K_B_* of region B at R = 0.1 had a better match with the $\Delta K_{eff}$ of the experimental value about FCG at 630 °C, the value of Δ*K_B_,* which was calculated by the tensile stress can be used to stand for the value of $\Delta K_{eff}$. Since the local yield stress $\left(\sigma_{y}\right)$ of the crack tip is different in air and vacuum [21], and it is hard for $\sigma_{y}$ to measure in the vacuum environment. Thus, the $\sigma_{y}$ in air under 630 °C was selected. In contrast to this, Δ*K* of Equation (14) should choose the data of the surface-induced fracture. Thus, the calculated value of $\gamma_{p}$ was 21.5 µm at 630 °C. Since the cycle plastic zone ($\gamma_{p}$) of the crack tip reached the mean size of the grain, the fracture morphology and crack growth rate will change. Meanwhile, the mean size of the grain was constant for the same material. Thus, the $\sigma_{y}$ in the vacuum environment could be calculated by the Δ*K_B_* of interior-induced cracks. So, the crack threshold formula about interior-induced fracture was proposed based on the Equations (3) and (14) for R = −1 and 0.1.

(15) $$\Delta K_{\mathrm{th},\mathrm{internal}}=\alpha \Delta K_{B,\mathrm{internal}}\sqrt{\frac{0.47d}{\gamma_{p}(1-\upsilon^{2})}}$$

where, Δ*K_B_*_,internal_ is the mean value of stress intensity factor of the region (B) on the interior-induced fracture; α is a parameter about stress ratios; *γ_p_* is the cycle plastic zone of the crack tip; d is the grain size; v is the Poisson’s ratio; and, based on the previous study, $\Delta K_{th,\mathrm{internal}}$ is positively related to the $\Delta K_{B,\;\mathrm{internal}}\sqrt{\frac{0.47d}{\gamma_{p}\left(1-v^{2}\right)}}$. Thus, the parameter α can be obtained by the least square method; when R = −1, α is 3.861 and when R = 0.1, α is 5.488. In order to check the accuracy of the predicted crack growth threshold on the interior-induced fracture ($\Delta K_{th,\mathrm{internal}}$) using Equation (15) at R = −1 and 0.1, the statistical analysis is presented in Figure 17. It indicates that the predicted data by Equation (15) had good accuracy, so Equation (15) could be used to predict the crack growth threshold of 9%Cr.

## 4. Conclusions

This study focused on the effect of the stress ratios on very high cycle fatigue behavior of 9%Cr turbine steel at 630 °C. The mechanical properties of 9%Cr in different environments were tested. For the fatigue test, two failure modes were observed at different stress ratios, including surface crack failure and internal crack failure, respectively. Besides, a new model for the crack growth threshold of the interior-induced fracture at different stress ratios was proposed and discussed.

(1) Through the uniaxial tensile test, it could be known that the material had a serious softening phenomenon at 630 °C.
(2) The S–N curves presented a continuous downward trend and had no fatigue limit. Under the high temperature environment, the fatigue strength of the specimen surface was reduced.
(3) For R = −1, the specimens were more likely to fail on the surface due to the decrease of the surface strength under high temperature. For R = 0.1, the specimen was more likely to exhibit internal failure behavior.
(4) For R = −1, the area of the region (A) and (B) of internal failures increased with the increase of the fatigue life. For R = 0.1, the area of about region (A) and (B) of internal failures decreased with the increase of the fatigue life.
(5) For R = −1, Δ*K_B_* about region (B) was close to a constant and had no obvious change with the increase of the fatigue life. For R = 0.1, Δ*K_B_* of internal failures regions (B) decreased with the increase of the fatigue life.
(6) The formula about the crack growth threshold of the interior-induced fracture was proposed and discussed, and the statistical analysis was used to check the difference and accuracy of data.

## Figures and Tables

**Table 1:** Chemical compositions of 9%Cr turbine steel (wt %).

C	Si	Mn	Cr	Mo	Ni	Nb	W	Co	B	N
0.12	0.06	0.20	9.16	0.20	0.40	0.08	2.95	2.82	0.007	0.02

**Table 2:** Tensile properties of 9%Cr turbine steel at RT and 630 °C.

Temperature	Elastic Modulus (GPa)	Yield Stress (MPa)	Tensile Strength (MPa)	K_1_ (MPa)	n_1_
RT	260.78	678.68	885	1141.39	0.078
630 °C	148.97	443.67	507.5	459.44	0.02

**Table 3:** Experimental and theoretical effective thresholds.

	$\Delta K_{\mathrm{th}}\;\mathrm{of}\;\mathrm{Surface}-\mathrm{Induced}\;(MPa\sqrt{m})$		$\Delta K_{\mathrm{th}}\;\mathrm{of}\;\mathrm{Interior}-\mathrm{Induced}\;(MPa\sqrt{m})$	
Stress Ratios	Experimental Value	Theoretical Value	Experimental Value	Theoretical Value
**R = −1**	1.56	1.38	1.88	1.38
**R = 0.1**	1.8	1.84	2.86	1.84

## References

[1] Rojas D., Garcia J., Prat O., Sauthoff G., Kaysser-Pyzalla A.R. (2011). 9%Cr heat resistant steels: Alloy design, microstructure evolution and creep response at 650 °C. Mater. Sci. Eng. A.

[2] He J.J., Chen J., Sun Q.M. (2014). Effect of Loading Rate on Low-Cycle Fatigue Properties of Turbine Rotor Steel. Procedia Mater. Sci..

[3] Zhu X., Chen H., Xuan F., Chen X. (2019). On the creep fatigue and creep rupture behaviours of 9–12% Cr steam turbine rotor. Eur. J. Mech. A Solids.

[4] Fournier B., Dalle F., Sauzay M., Longour J., Salvi M., Caes C., Tournié I., Giroux P.-F., Kim S.-H. (2011). Comparison of various 9–12%Cr steels under fatigue and creep-fatigue loadings at high temperature. Mater. Sci. Eng. A.

[5] Wu Q.J., Lu F.G., Cui H.C., Ding Y.M., Liu X., Gao Y.L. (2014). Microstructure characteristics and temperature-dependent high cycle fatigue behavior of advanced 9% Cr/CrMoV dissimilarly welded joint. Mater. Sci. Eng. A.

[6] Zhang Q., Zhang J., Zhao P., Huang Y., Yu Z., Fang X. (2016). Low-cycle fatigue behaviors of a new type of 10% Cr martensitic steel and welded joint with Ni-based weld metal. Int. J. Fatigue.

[7] Mishnev R., Dudova N., Kaibyshev R. (2020). Effect of microstructural evolution on the cyclic softening of a 10% Cr martensitic steel under low cycle fatigue at 600 °C. Int. J. Fatigue.

[8] Rae Y., Guo X., Benaarbia A., Neate N., Sun W. (2020). On the microstructural evolution in 12% Cr turbine steel during low cycle fatigue at elevated temperature. Mater. Sci. Eng. A.

[9] Zhang W., Wang X.W., Chen H.F., Zhang T.Y., Gong J.M. (2019). Evaluation of the effect of various prior creep-fatigue interaction damages on subsequent tensile and creep properties of 9%Cr steel. Int. J. Fatigue.

[10] Yan Y.M., Hu Z.F., Lin F.S., Zhao S.Q. (2012). Fatigue behavior of 30Cr1Mo1V rotor steel at elevated temperature after long-term service. J. Mater. Eng..

[11] Ziegler D., Puccinelli M., Bergallo B., Picasso A. (2013). Investigation of turbine blade failure in a thermal power plant. Case Stud. Eng. Fail. Anal..

[12] Hui W., Zhang Y., Zhao X., Chao Z., Wang K., We S., Dong H. (2016). Very high cycle fatigue properties of Cr-Mo low alloy steel containing V-rich MC type carbides. Mater. Sci. Eng. A.

[13] Zhu M.-L., Xuan F.-Z., Chen J. (2012). Influence of microstructure and microdefects on long-term fatigue behavior of a Cr–Mo–V steel. Mater. Sci. Eng. A.

[14] Zhu M.-L., Liu L.-L., Xuan F.-Z. (2015). Effect of frequency on very high cycle fatigue behavior of a low strength Cr–Ni–Mo–V steel welded joint. Int. J. Fatigue.

[15] Hong Y., Lei Z., Sun C., Zhao A. (2014). Propensities of crack interior initiation and early growth for very-high-cycle fatigue of high strength steels. Int. J. Fatigue.

[16] Zhang W.-C., Zhu M.-L., Wang K., Xuan F.-Z. (2018). Failure mechanisms and design of dissimilar welds of 9%Cr and CrMoV steels up to very high cycle fatigue regime. Int. J. Fatigue.

[17] Hilgendorff P.M., Grigorescu A.C., Zimmermann M., Fritzen C.P., Christ H.J. (2016). Cyclic deformation behavior of austenitic Cr-Ni-steels in the VHCF regime: Part II—Microstructure-sensitive simulation. Int. J. Fatigue.

[18] Kovacs S., Beck T., Singheiser L. (2013). Influence of mean stresses on fatigue life and damage of a turbine blade steel in the VHCF-regime. Int. J. Fatigue.

[19] Grigorescu A.C., Hilgendorff P.M., Zimmermann M., Fritzen C.P., Christ H.J. (2016). Cyclic deformation behavior of austenitic Cr–Ni-steels in the VHCF regime: Part I – Experimental study. Int. J. Fatigue.

[20] Milošević I., Renhart P., Winter G., Grün F., Kober M. (2018). Validation of a new high frequency testing technique in the VHCF regime–Fatigue properties of a 42CrMoS4 and X5CrNiCuNb16–4 steel. Int. J. Fatigue.

[21] Yang K., He C., Huang Q., Huang Z.Y., Wang C., Wang Q., Liu Y.J., Zhong B. (2017). Very high cycle fatigue behaviors of a turbine engine blade alloy at various stress ratios. Int. J. Fatigue.

[22] (2011). ASTM. Standard Test Method for Measurement of Fatigue Crack Growth Rates.

[23] Zhu M.-L., Xuan F.-Z., Tu S.-T. (2013). Interpreting load ratio dependence of near-threshold fatigue crack growth by a new crack closure model. Int. J. Press. Vessel. Pip..

[24] El Rayes M.M., El-Danaf E.A. (2017). High temperature deformation behavior of as-produced and retired 9–12% Cr power plant steel. Mater. Sci. Eng. A.

[25] He J.L., Chen F., Wang B., Zhu L.B. (2018). A modified Johnson-Cook model for 10%Cr steel at elevated temperatures and a wide range of strain rates. Mater. Sci. Eng. A.

[26] Sakai T., Sato Y., Nagano Y., Takeda M., Oguma N. (2006). Effect of stress ratio on long life fatigue behavior of high carbon chromium bearing steel under axial loading. Int. J. Fatigue.

[27] Hou Fang J.L., Shaoxiong X., Yongjie L., Qingyuan W., Junhui Z. (2016). Very high cycle fatigue properties of CrMoW rotor steel at high-temperature. J. Mater. Res..

[28] Hong Y., Liu X., Lei Z., Sun C. (2016). The formation mechanism of characteristic region at crack initiation for very-high-cycle fatigue of high-strength steels. Int. J. Fatigue.

[29] Sakai T., Sato Y., Oguma N. (2001). Characteristic S-N Property of High Carbon Chromium Bearing Steel under Axial Loading in Long Life Fatigue. Nihon Kikai Gakkai Ronbunshu A Hen/ Trans. Jpn. Soc. Mech. Eng. A.

[30] Sakai T., Tanaka N., Takeda M., Kanemitsu M., Oguma N. Characteristic S-N property of high strength steels in ultra-wide life region under rotating bending. Proceedings of the Second International Symposium on Environmentally Conscious Design and Inverse Manufacturing.

[31] Murakami Y. (1990). Effects of small defects and nonmetallic inclusions on the fatigue strength of metals. Key Eng. Mater..

[32] Yang K., Zhong B., Huang Q., He C., Huang Z.Y., Wang Q., Liu Y.-J. (2018). Stress Ratio and Notch Effects on the Very High Cycle Fatigue Properties of a Near-Alpha Titanium Alloy. Materials.

[33] Chen Y., He C., Liu F., Wang C., Liu Y. (2019). Effect of microstructure inhomogeneity and crack initiation environment on the very high cycle fatigue behavior of a magnesium alloy. Int. J. Fatigue.

[34] Taylor D. (1989). Fatigue Thresholds.

[35] Pokluda J., Pippan R., Vojtek T., Hohenwarter A. (2014). Near-threshold behaviour of shear-mode fatigue cracks in metallic materials. Fatigue Fract. Eng. Mater. Struct..

[36] Xu D.K., Liu L., Xu Y.B., Han E.H. (2007). The crack initiation mechanism of the forged Mg–Zn–Y–Zr alloy in the super-long fatigue life regime. Scr. Mater..

[37] Zhao A., Xie J., Sun C., Lei Z., Hong Y. (2011). Prediction of threshold value for FGA formation. Mater. Sci. Eng. A.

[38] Oguma H., Nakamura T. (2013). Fatigue crack propagation properties of Ti-6A1-4V in vacuum environments. Int. J. Fatigue.

[39] Stanzl-Tschegg S., Schonbauer B., Lukas P. (2010). Near-threshold fatigue crack propagation and internal cracks in steel. Fatigue.

[40] Kobayashi A.S., Murakami Y., Hasebe M.T., Itoh Y., Kishimoto K., Miyata H., Miyazaki N., Terada H., Tohgo K., Yuuki R. (2008). Stress Intensity Factors Handbook.

[41] Liu F., He C., Chen Y., Zhang H., Wang Q., Liu Y. (2020). Effects of defects on tensile and fatigue behaviors of selective laser melted titanium alloy in very high cycle regime. Int. J. Fatigue..

[42] Uwe Z., Vormwald M., Pippan R., Gänser H.-P., Sarrazin-Baudoux C., Madia M. (2016). About the fatigue crack propagation threshold of metals as a design criterion—A review. Eng. Fract. Mech..

[43] Paris P., Erdogan F. (1963). A Critical Analysis of Crack Propagation Laws. J. Basic Eng. Fail. Anal..

[44] Elber W., Rosenfeld M. (1971). The significance of fatigue crack closure. Damage Tolerance in Aircraft Structures.

[45] Duquette D.J., Gell M. (1971). The Effect of Environment on the Mechanism of Stage I Fatigue Fracture. Metall. Trans..

[46] Yuen A., Hopkin S.W., Leverant G.R., Rau C.A. (1974). Correlations between fracture surface appearance and fracture mechanics parameters for stage II fatigue crack propagation in TÏ-6AI-4V. Metall. Trans..

